# Evaluation for the Clinical Diagnosis of *Pythium insidiosum* Using a Single-Tube Nested PCR

**DOI:** 10.1007/s11046-013-9695-3

**Published:** 2013-08-15

**Authors:** Yordhathai Thongsri, Lumyai Wonglakorn, Angkana Chaiprasert, Lucie Svobodova, Petr Hamal, Maitree Pakarasang, Chularut Prariyachatigul

**Affiliations:** 1 Faculty of Graduated School, Khon Kaen University, Khon Kaen, Thailand; 2 Microbiology Laboratory, Faculty of Medicine Srinagarind Hospital, Khon Kaen University, Khon Kaen, Thailand; 3 Department of Microbiology, Faculty of Medicine Siriraj Hospital, Mahidol University, Bangkok, Thailand; 4 Department of Microbiology, Faculty of Medicine and Dentistry, Palacky University and University Hospital Olomouc, Olomouc, Czech Republic; 5 Centre for Research and Development of Medical Diagnostic Laboratories, Department of Microbiology, Faculty of Associated Medical Sciences, Khon Kaen University, Khon Kaen, 40002 Thailand

**Keywords:** *Pythium insidiosum*, Pythiosis, Single-tube nested PCR, 18S rRNA

## Abstract

Pythiosis is a rare infectious disease caused by *Pythium insidiosum,* which typically occurs in tropical and subtropical regions. The high mortality rate may be in consequence of the lack of diagnosis. The objective of this study was to evaluate reliability of a new single-tube nested PCR for detection of *P. insidiosum* DNA. A total of 78 clinical isolates of various fungi and bacteria, 106 clinical specimens and 80 simulated positive blood samples were tested. The developed primer pairs CPL6–CPR8 and YTL1–YTR1 are located on 18S subunit of the rRNA gene of *P. insidiosum*. The specificity, negative and positive predictive values were 100, 100 and 87.5 %, respectively, as compared with direct microscopy and cultivation. The detection limit of the single-tube nested PCR was 21 zoospores corresponding to 2.7 pg of the DNA. The results demonstrate that the new single-tube nested PCR offers a highly sensitive, specific and rapid genetic method for detecting *P. insidiosum.*

## Introduction

Pythiosis is an emerging and life-threatening infectious disease caused by the oomycete *Pythium insidiosum,* which can develop in humans and animals [1–4]. The infective stage of *P. insidiosum* is a biflagellate zoospore [1]. Human pythiosis occurs predominantly in tropical areas of the world and particularly in Thailand [1, 5, 6]. There, the first documented case of human pythiosis was reported in 1985 [7]. According to clinical signs, human pythiosis can be divided into four types: cutaneous/subcutaneous, vascular, ocular and disseminated. The first type is characterized by chronic swelling, painful subcutaneous granulomatous infiltration and ulceration, usually located in the face or legs [8]. Chronic arthritis in the lower extremities resulting in arterial occlusion and gangrenous ulceration of feet or legs is typical for vascular pythiosis [9]. The ocular form is usually manifested as corneal ulcers or keratitis. As a result of all these forms of infection, *P. insidiosum* can spread via the bloodstream to various internal organs or organ systems such as the gastrointestinal tract, brain, liver, kidney or rhinosinus [10]. Currently, the diagnosis of pythiosis is based on microscopy, culture, detection of antibodies and molecular genetic techniques [9, 11–13]. However, microscopy cannot distinguish zygomycetes because of the coenocytic form of the mycelium [7]. Culture is time-consuming, and obtaining infected tissue samples may be difficult [1]. Because of low antibody response, false-negative results frequently occur in serological tests, particularly in ocular pythiosis [11]. Nested PCR has been developed for the diagnosis of pythiosis using the internal transcribed spacer 1 (ITS1) of the gene for rRNA [14]. Although it is highly sensitive, the main problem of this PCR is a high risk of contamination as the product of the first reaction needs to be transferred into another tube for the second reaction. The purpose of this study was to solve this problem by developing a nested PCR for detecting *P. insidiosum* in a single tube and to evaluate its reliability using various clinical specimens, including simulated positive blood samples and clinical isolates of bacteria and fungi.

## Materials and Methods

### Clinical Isolates

The study comprised 34 isolates of *P. insidiosum* as specified in Table 1, 29 fungal isolates (*Aspergillus fumigatus*, *Aspergillus flavus*, *Aspergillus niger,* *Basidiobolus ranarum, Candida albicans*, *Candida tropicalis*, *Cladophialophora carrionii*, *Curvularia* spp*., Exophiala jeanselmei*, *Filobasidiella neoformans*, *Fusarium* spp., *Hortaea werneckii*, *Lichtheimia* spp., *Microsporum gypseum*, *Mucor* spp., *Penicillium* spp., *Rhodotorula* spp., *Rhizopus* spp., *Saccharomyces cerevisiae*, *Scedosporium apiospermum*, *Syncephalastrum* spp., *Talaromyces marneffei*, *Trichophyton* spp., *Trichophyton concentricum, Trichophyton rubrum*, *Trichophyton schoenleinii*, *Trichophyton tonsurans, Trichophyton violaceum* and *Trichosporon* spp.), 10 bacterial isolates (*Burkholderia pseudomallei*, *Corynebacterium* spp., *Enterococcus* spp., *Escherichia coli*, *Klebsiella pneumoniae*, *Pseudomonas aeruginosa*, *Salmonella typhi*, *Staphylococcus aureus, Streptococcus pyogenes* and *Streptococcus viridans*) and 5 isolates of non-*insidiosum Pythium* spp. (*P. aphanidermatum*, *P. deliense*, *P. grandisporangium*, *P. middletonii* and *P. ultimum*). All microorganisms were obtained from patients of the Siriraj Hospital, Mahidol University, Bangkok, Thailand. All isolates were identified based on conventional microbiological methods and then stored in skim milk (Oxoid, UK) 100 mg/ml with 33 % glycerol at −20 °C.

**Table 1:** Sources of *P. insidiosum* strains

Reference no.	Clinical form of pythiosis	Patient information		
		Age/sex	Years	Residential province (Thailand)
1. CBS 673.85	Cutaneous	23/M	1985	NA
2. MCC 1	Ocular	20/M	1989	Samut Prakan
3. MCC 2	Ocular	33/F	1992	Chai Nat
4. MCC 3	Cutaneous	40/M	1991	Phichit
5. MCC 4	Vascular	25/M	1986	Suphan Buri
6. MCC 5	Vascular	49/F	1993	Bangkok
7. MCC 6	Ocular	NA	NA	Ayutthaya
8. MCC 7	Ocular	62/M	NA	Ayutthaya
9. MCC 8	Ocular	62/M	NA	Narathiwat
10. MCC 9	Vascular	46/F	1986	Phichit
11. MCC 10	Disseminated	12/M	2000	Saraburi
12. MCC 11	Ocular	48/F	NA	Chon Buri
13. MCC 29	Disseminated	NA	NA	Ratchaburi
14. SIMI 10201	Ocular	23/F	1989	Phatthalung
15. SIMI 149-41	Vascular	14/M	1988	Lop Buri
16. SIMI 16068	Ocular	26/F	1994	NA
17. SIMI 1839-46	Vascular	19/F	2003	Nakhon Pathom
18. SIMI 18093	Ocular	50/F	1995	Samut Sakhon
19. SIMI 240-37	Ocular	NA/M	1994	Samut Sakhon
20. SIMI 283-40	Ocular	58/F	1997	Bangkok
21. SIMI 2921-45	Ocular	75/F	2002	Ratchaburi
22. SIMI 2989-42	Vascular	72/F	1999	Suphan Buri
23. SIMI 322-37	Ocular	26/M	1994	NA
24. SIMI 3306-44	Ocular	NA/F	2001	Nakhon Si Thammarat
25. SIMI 348-37	Ocular	36/M	1994	Bangkok
26. SIMI 4523-45	Ocular (corneal transplantation)	37/M	2002	Pathum Thani
27. SIMI 4763	Cutaneous	40/M	2001	Ratchaburi
28. SIMI 6666	Ocular	42/M	1986	Kamphaeng Phet
29. SIMI 7873	Vascular	31/F	1988	Chanthaburi
30. SIMI 7874	Vascular	31/F	1988	Chanthaburi
31. SIMI 8659	Vascular	52/M	1988	Suphan Buri
32. SIMI 8727	Vascular	19/M	1988	Yasothon
33. SIMI 9642	Ocular	53/F	1989	Yasothon
34. SIMI 9743	Ocular	53/F	1989	Yasothon

*CBS* Centralbureau voor Schimmelcultures, Utrecht, The Netherlands; *MCC* mycology culture collection at the Department of Microbiology, Mahidol University; *SIMI* microbial culture collection at the Siriraj Hospital Microbiology Laboratory, Mahidol University, Bangkok, Thailand; *NA* data not available; *M* male; *F* female

### Clinical Specimens

One hundred and six clinical specimens from patients with suspected fungal infection obtained from a routine mycology laboratory in the Srinagarind Hospital, Khon Kaen University, Khon Kaen, Thailand were evaluated prospectively from May 2011 to February 2012. They included pus (*n* = 27), tissue biopsies (*n* = 19), blood (*n* = 10), bone marrow (*n* = 9), lymph nodes (*n* = 6), peritoneal dialysis fluid (*n* = 6), sputum (*n* = 5), bronchial washing (*n* = 5), tracheal secretion (*n* = 5), urine (*n* = 5), cerebrospinal fluid (*n* = 4), synovial fluid (*n* = 2), ascitic fluid (*n* = 2) and pleural fluid (*n* = 1). In addition, 80 simulated positive blood specimens were prepared from normal blood samples. For this purpose, each blood sample was mixed with zoospores of *P. insidiosum* so that each sample contained 1.15 × 10^6^ zoospores ml/l.

### Evaluation by Phenotypic Methods

All clinical specimens were evaluated microscopically in 20 % potassium hydroxide preparation for the presence of *P. insidiosum* hyphae. Culture was performed, with each specimen being inoculated on two Sabouraud dextrose agars (SDA; Oxoid, UK), two Mycosel agars (MCA; BD Diagnostics) and one blood agar (Oxoid, UK) for detection of *P. insidiosum* growth. One SDA and MCA each were incubated at 25 °C and the other media at 37 °C. All agars were evaluated for the *P. insidiosum* growth until 30 days. The suspected colonies were identified as *P. insidiosum* by induction of zoospores [15]. Results of these phenotypic methods were then compared with a single-tube nested PCR for sensitivity, specificity, positive predictive value (PPV) and negative predictive value (NPV).

### DNA Extraction for the PCR

DNA from all clinical and simulated positive specimens was extracted with the NucleoSpin Tissue kit (Macherey–Nagel, Germany) and QIAamp DNA Mini Kit (Qiagen) according to the manufacturers’ instructions. All fungal isolates were cultured in 250 ml of Sabouraud dextrose broth (Oxoid, UK) and incubated at a room temperature for 7 days with shaking (150 rpm) in a rotary shaker (PSU 2T plus, BioSan, Latvia). Fungal mycelia were filtered, washed twice with deionized water and frozen at −20 °C until used. Bacterial isolates were cultured in 3 ml of Luria broth (Oxoid, UK) and incubated at 37 °C for 16–18 h with shaking (200 rpm). Then, cultures were transferred to a 1.5 ml microtube, centrifuged and stored at 4 °C. For cell disruption, approximately 30 mg of frozen fungal mycelia and 0.14–1.21 g of the bacterial pellet were rubbed in liquid nitrogen until a fine powder. The bacterial powder was then suspended in a lysis buffer (25 mM Tris–HCl pH 8, 10 mM EDTA pH 8, 100 mM NaCl). DNA was extracted from the bacterial suspensions and powders from fungal mycelia as described by Sambrook and Russell [16]. Purity of isolated DNA was calculated using a spectrophotometer at OD 260 and 280.

### Single-Tube Nested PCR

**Fig. 1:**
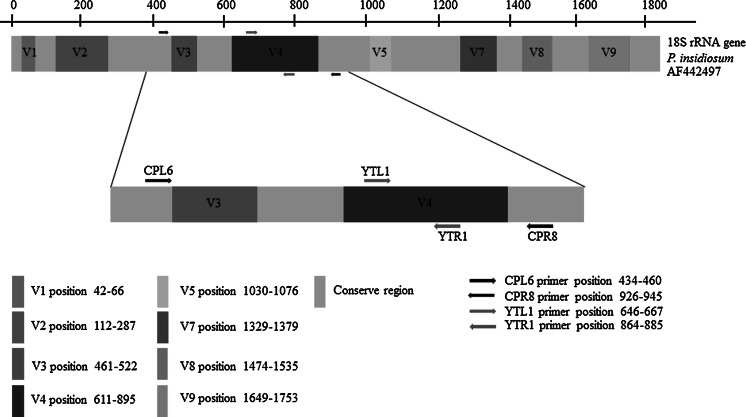
Position of the annealing sites of the primer pairs CPL6–CPR8 and YTL1–YTR1 in the 18S rRNA gene of *P. insidiosum*

The single-tube nested PCR was performed using outer primers CPL6 (5′-GAC ACA GGG AGG TAG TGA CAA TAA ATA-3′) and CPR8 (5′-CTT GGT AAA TGC TTT CGC CT-3′), and inner primers YTL1 (5′-CTT TGA GTG TGT TGC TAG GAT G-3′) and YTR1 (5′-CTG GAA TAT GAA TAC CCC CAA C-3′) designed by ourselves using the GenBank database. The primers are located on the 18S subunit of the rRNA gene of *P. insidiosum* (GenBank accession no. AF442497). The position of the newly designed primer is shown in Fig. 1. The reaction mixture in a total volume of 50 μl contained 1 U of Platinum Taq DNA polymerase (Invitrogen), 0.2 mM MgCl_2_, 25 pmol of each primers and 0.2 mM dNTPs. Amplification was performed in a thermal cycler (Mastercycler Personal, Eppendorf International) using the following temperature cycles: initial denaturation at 95 °C for 5 min, followed by 30 cycles (denaturation at 95 °C for 1 min, annealing at 68 °C for 1 min and extension at 72 °C for 1 min), another 30 cycles (denaturation at 95 °C for 1 min, annealing at 57 °C for 1 min and extension at 72 °C for 1 min) and final extension for 10 min at 72 °C. Amplicons were visualized under UV light using a manual documentation system (InGenius3, Syngene, USA) after electrophoresis of 10 μl of the reaction solution in 2 % UltraClean agarose gel (Mo Bio Laboratories) containing the SYBR Gold nucleic acid gel stain (Invitrogen).

### Evaluation of PCR Sensitivity

To determine the lowest detection limit of our nested PCR, seven 10-fold dilutions were prepared from simulated positive blood specimen. Then, 180 μl of each dilution containing 2.07 × 10^5^–2.07 × 10^−1^ zoospores was used for nested PCR. As a control, DNA of *P. insidiosum* hyphae was also prepared as 10-fold serial dilution from 1 ng to 1 fg, and then, it was also amplified by a single-tube nested PCR. This study was approved by the Ethical Committee on Human Experimentation in Khon Kaen University in accordance with the Declaration of Helsinki (HE 541111).

## Results

If *P. insidiosum* DNA was presented in clinical material, four amplicons sized 512, 452, 340 and 240 bp were detected by our nested PCR.

### Evaluation of PCR Specificity Within Clinical Isolates

**Fig. 2:**
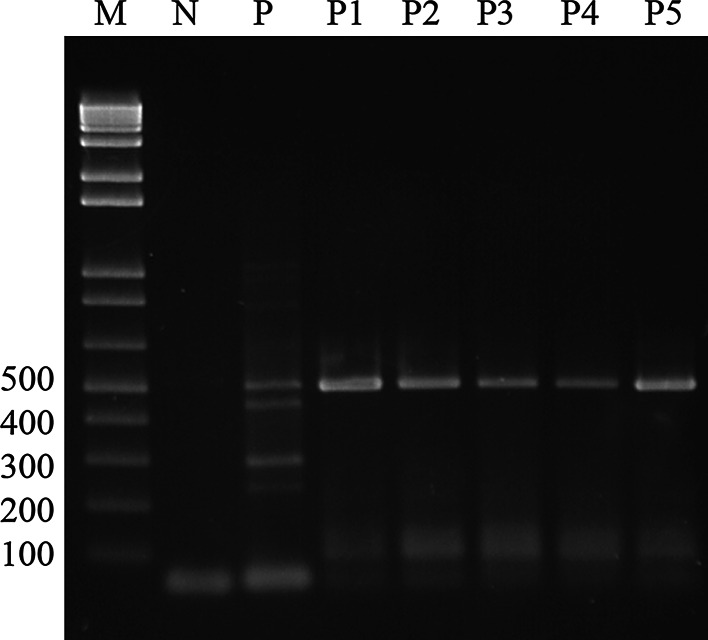
Specificity of the single-tube nested PCR for amplification of DNA from *Pythium* spp. *Lane M* 100 bp DNA ladder, *lane N* negative control, *lane P* *P. insidiosum*, *lane P1* *P. middletonii*, *lane P2* *P. ultimum*, *lane P3 P. aphanidermatum*, *lane P4 P. deliense*, *lane P5 P. grandisporangium*

Four amplicons were detected in all 34 *P. insidiosum* isolates. Only one genus-specific product (512 bp) was found in five non-*insidiosum Pythium* spp. as demonstrated in Fig. 2. No product was found after amplification of DNA from all the remaining 29 fungal and 10 bacterial isolates. Therefore, our single-tube nested PCR had 100 % specificity within the group of clinical microorganisms tested in this study.

### Evaluation of PCR Specificity in Clinical Specimens

Based on positivity of phenotypic methods, seven pus specimens from corneal ulcers were evaluated as positive within all tested clinical specimens. From that, three specimens were microscopically negative; culture and zoospore induction were positive in all seven specimens. Moreover, as demonstrated in Table 2, one more pus specimen, also from a corneal ulcer, which was negative in both phenotypic methods, was positive in our single-tube nested PCR.

**Table 2:** Comparison of *P. insidiosum* detection in positive clinical specimens by phenotypic methods and a single-tube nested PCR

Specimen no.	Phenotypic method		Genotypic method
	Microscopy	Culture and zoospore induction	Single-tube nested PCR
1	+	+	+
2	+	+	+
3	+	+	+
4	−	+	+
5	−	−	+
6	+	+	+
7	−	+	+
8	−	+	+

All specimens were pus from corneal ulcers

### Evaluation of PCR Sensitivity

**Fig. 3:**
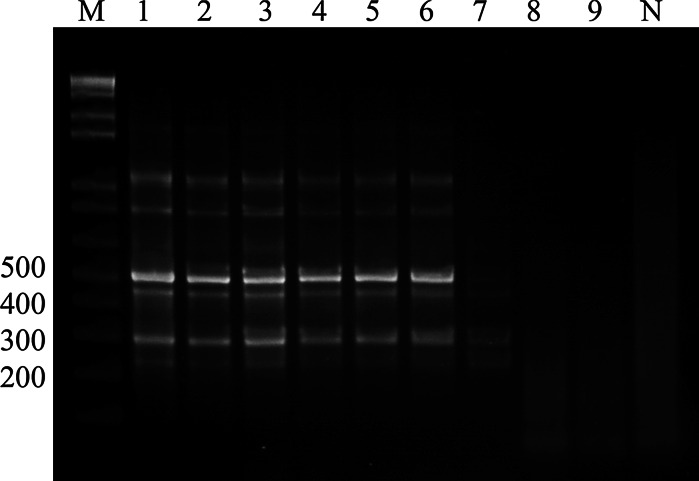
Sensitivity of the single-tube nested PCR for detection of *P. insidiosum* DNA from zoospores. *Lane M* 100 bp DNA ladder, *lane N* negative control, *lane 1* *P. insidiosum* DNA (positive control), *lane 2* 2.07 × 10^5^ zoospores in double distilled water (DDW), *lanes 3*–*9* dilution of simulated positive blood specimen in DDW (*lane 3* 2.07 × 10^5^ zoospores, *lane 4* 2.07 × 10^4^ zoospores, *lane 5* 2.07 × 10^3^ zoospores, *lane 6* 2.07 × 10^2^ zoospores, *lane 7* 2.07 × 10^1^ zoospores, *lane 8* 2.07 zoospores, *lane 9* 2.07 × 10^−1^ zoospores)

As shown in Fig. 3, the lowest detection limit in diluted simulated positive blood specimen was 21 zoospores, which corresponded to 2.7 pg of DNA. The lowest detection limit of serially diluted DNA isolated from *P. insidiosum* hyphae was 1 pg.

### Evaluation of PPV and NPV of PCR in Clinical Specimens

These results are summarized in Table 3. Based on results of phenotypic methods, 7 specimens were evaluated as positive for *P. insidiosum*. Using our single-tube nested PCR, DNA of *P. insidiosum* was detected in 8 specimens, whereas 7 of them were positive by phenotypic methods. Therefore, NPV and PPV were 100 and 87.5 %, respectively.

**Table 3:** Comparison of phenotypic test results with a single-tube nested PCR

	Phenotypic tests (microscopy in 20 % KOH and culture)		Total
	Positive	Negative	
Single-tube nested PCR			
Positive	7	1	8
Negative	0	98	98
Total	7	99	106

## Discussion

In our set of clinical specimens, one patient was found to be positive in single-tube nested PCR, but negative in phenotypic tests. In our study, the criterion for detecting pythiosis was positivity in one of two phenotypic methods. However, Krajaejun et al. [9] defined the criteria for pythiosis as culture positivity, seropositivity or clinical manifestation. The patient who had negative results of phenotypic tests and was positive in nested PCR had symptoms of ocular pythiosis evaluated by a clinician. The reason why phenotypic methods could not confirm pythiosis in this case was probably their low sensitivity [17]. So, this finding demonstrates the superiority of PCR over phenotypic methods from the sensitivity point of view. The detection limit of our nested PCR in this study (2.7 pg of DNA in relation to 21 counted zoospores) allows us to suppose that one zoospore contains 128.57 fg of DNA. However, our experiments showed that the lowest detected amount of DNA from *P. insidiosum* isolated from its mycelium was 1 pg of DNA, corresponding to about eight zoospores. According to this detection limit, one zoospore could contain 48–129 fg of DNA. This is the first report which tried to estimate the amount of DNA in *P. insidiosum* zoospores. Hussain et al. [18] reported a detection limit of PCR for *Phytophthora infestans* of 0.5 pg, which corresponded to four zoospores; in that case, one zoospore should contain 125 fg of DNA. The DNA content is similar to data about plant pathogenic organisms from the order *Peronosporales* (*Oomycota*) ranging from 46 to 163 fg [19]. The newly designed, genus-specific outer set of primers CPL6 and CPR8 amplified a 512 bp fragment from a conserved part of the 18S subunit of the rRNA gene and species-specific inner primers YTL1 and YTR1 amplified a 240 bp fragment from variable region of this subunit were firstly evaluated in this study. Although finally four PCR products were seen in a single-tube nested PCR, the test showed excellent sensitivity and specificity. The fragment of 452 bp was the product of CPL6 and YTR1 primers, and the 300 bp fragment was amplified by the YTL1 and CPR8 primers. Four PCR products were detected in all 34 strains of *P. insidiosum* and in clinical samples from patients with pythiosis. A sensitivity of 100 % and a low detection limit allow us to recommend our single-tube nested PCR as a useful tool for laboratories, which need to detect *P. insidiosum* in clinical material. Pythiosis is a rare but fatal infection in humans. Krajaejun et al. [8] reported 102 cases of human pythiosis in Thailand from 1985 to 2003. The zoospore is the infective stage of *P. insidiosum*; therefore, the selected spiking of blood with zoospores simulates the state in really infected blood better than spiking it with pure DNA. The sensitivity of our nested PCR tested on 78 various clinical isolates and 186 clinical specimens including simulated positive blood samples was higher than with other tests [20–22]. In addition, nested PCR solves the main drawbacks of other methods for detecting pythiosis. It overcomes problems with culture and allows identification in a substantially shorter time directly in clinical specimens. Thus, the prognosis of patients can be improved because treatment can be started earlier. Moreover, performing nested PCR in a single tube markedly reduces the risk of cross-contamination. Results of this study demonstrate that our single-tube nested PCR is suitable for detecting *P. insidiosum* directly from clinical specimens and identification of clinical isolates as well. As this method is rapid, highly sensitive and has minimal risk of cross-contamination, it has a great potential in the diagnosis of suspected cases of pythiosis.
